# Characterization of Population Genetic Structure of red swamp crayfish, *Procambarus clarkii*, in China

**DOI:** 10.1038/s41598-018-23986-z

**Published:** 2018-04-03

**Authors:** Shaokui Yi, Yanhe Li, Linlin Shi, Long Zhang, Qingbin Li, Jing Chen

**Affiliations:** 1 College of fisheries, Key Lab of Agricultural Animal Genetics, Breeding and Reproduction of Ministry of Education, Huazhong Agricultural University, Wuhan, 430070 P. R. China; 2 Fish Genetics and Breeding Laboratory, The Ohio State University South Centers, Piketon, 45661 United States of America; 3 Institute of Fisheries, Anhui Academy of Agricultural Sciences, Hefei, 230031 P. R. China

## Abstract

The red swamp crayfish (*Procambarus clarkii*) is one of the most economically important farmed aquatic species in China. However, it is also a famous invasive species in the world. This invasive species was dispersed most via human activities including intentional or unintentional carry in China. Thus, *P. clarkii* naturally distributed in China provides us a desirable mode to investigate the genetic structure of an invasive species dispersed mainly by human-mediated factors. To reveal the impact of human-mediated dispersal on genetic structure of *P. clarkii* in China, a total of 22,043 genome-wide SNPs were obtained from approximately 7.4 billion raw reads using 2b-RAD technique in this study. An evident pattern of population genetic structure and the asymmetrical migrational rates between different regions were observed with 22 populations based on these SNPs. This study provide a better understanding of the population genetic structure and demographic history of *P*. *clarkii* populations in China, inferring that anthropogenic factors (aquaculture or by accident) and ecological factors (e.g., complicated topography and climatic environment), as well as its special biological traits could account for the current population structure pattern and dispersal history of *P*. *clarkii*.

## Introduction

The red swamp crayfish, *Procambarus clarkii* (Girard 1985), native to north-eastern Mexico and south-central United States, is one of the world’s most invasive species^1,2^, and its aggressive burrowing leads to the damages of levee, dam and paddy field^3^. In 1920s, *P. clarkii* invaded China from Japan and now widely distributed in almost all types of freshwater habitats, e.g., swamps, sloughs, ditches and paddy fields, in China. Several traits of its life history, e.g., polytrophism, rapid growth, high fecundity and disease resistance, make its invasion of the wild successful^4^. Interestingly, it is widely favored and consumed in China, and is one of the most economically important farmed aquatic species rather than a devastating invasive species. In the cities located in the middle and lower reaches of the Yangtze River, annual consumption of *P. clarkii* reaches more than 600,000 metric tons during the peak summer season (www.yyj.moa.gov.cn). Thus, recently, much attention has been paid to the cultivation technique^5^, artificial reproduction^6^, and antibacterial or antiviral mechanisms of *P. clarkii*^7,8^. The dispersal rate of *P. clarkii* appeared to greatly exceed the estimated rates, and now it has invaded into most areas in China except Tibet plateau. Study on the population genetic structure of this alien species would contribute to understanding its biological invasions and establishing possible methodologies for its prevention and control^9^. Human-mediated jump dispersal of *P. clarkii* including intentional or unintentional carry, combined with its natural expansion influenced the population structure and genetic diversity^10,11^. Especially, with *P. clarkii* consumption demand increasing in recent years, the crayfish was carried into many areas by commercial transportation. The wild crayfish populations were mixed with the crayfish escaped from cultured population due to the natural disaster or human negligence. Therefore, the genetic structure of *P. clarkii* populations in China is complicated. Information on genetic structure and diversities of *P. clarkii* populations, which are mainly dispersed by human-mediated factors, would be more helpful to find a way to control its invasion and scientifically utilize the germplasm resources. Undoubtedly, *P. clarkii* could provide a desirable mode for investigating the genetic structure of an invasive species mainly dispersed by human-mediated factors. Previous studies^6,12,13^ proposed that the populations of *P. clarkii* in China exhibited a high level of genetic diversity after successfully invading a new environment. Meanwhile, in the past years, the human-mediated dispersal in aquaculture accelerated the invasion of *P. clarkii* in China and might result in the differnet  levels of gene flow among different populations^11^. However, the impacts of anthropogenic factors on the population structure of *P. clarkii* need to be comprehensively assessed, and the population genetic features of *P. clarkii* have changed during the past decades after the invasion. Therefore, the dynamics of population genetics in *P. clarkii* should be evaluated with a powerful method. The population structure and genetic diversity of this alien species help us revealing the historical dispersal pattern of *P. clarkii* in China, and also provide new insights into invasion prevention and utilization of genetic resource. Previously, population genetic studies of *P. clarkii* have generally used mitochondrial genes^12^, microsatellites^14,15^ and AFLP^13^, and that the genomic data of *P. clarkii* is quite limited^8,16^. The growing accessibility to high-throughput sequencing technologies allows the production of massive data and the discovery of genome-wide resources at relatively modest and decreasing costs. As a simple and flexible technique for genome-wide genotyping, 2b-RAD method can provide the rapid discovery of thousands of SNPs that are to some extent evenly distributed across the genome^17,18^. To date, to our knowledge, there is no document about investigating the population structure and genetic differentiation of *P. clarkii* at genome level after its successful invasion.

Recently, rapid adaptive evolution of invasive species has been a hot topic^19,20^. Theoretical considerations indicate that invading populations should be prime candidates for both adaptive and non-adaptive evolutionary change^21^. It is clear that invasion can drive evolutionary changes in the phenotypic traits of invasive organisms within periods of years to decades. To adapt the environmental conditions of its introduced area, invasive species rapidly evolved adaptive clines in key morphological and life-history characteristics after its invasion. Presently, most researches in this area tended to concentrate on comparing genetic diversity between native and invasive populations. Few studies have investigated the phenotypic divergence of the invasive animal species after its invasion. Zhang *et al*.^22^ firstly reported that the morphological differentiation of 12 different populations in *P. clarkii* and proposed the Chinese populations exhibited phenotypic variations (e.g., carapace, abdominal, body length) compared to American populations. Based on the basis of that previous study, the investigation of morphological characters between different populations of *P. clarkii* could help us understanding the rapid evolution of phenotypic traits of *P. clarkii* after its invasion.

In this study, amounts of genome-wide SNPs were identified in *P. clarkii* using 2b-RAD technique and then used into clarifying the genetic structure of *P. clarkii* in the main distribution areas in China. The genetic dynamics and genetic structure impacted by human-mediated dispersal, and phenotypic divergence of *P. clarkii* in China, would be presented in the study. We investigated the population genetic signatures and conducted morphological analyses to test the hypotheses that (1) the *P. clarkii* populations impacted by anthropogenic factors show persistent asymmetrical gene flow and exhibit an evident pattern of genetic structure; (2) rapid evolution or selection affects the phenotypic divergence of different *P. clarkii* populations. These results would contribute to exhibiting the genetic signatures of *P. clarkii* populations after the successful invasion, providing management strategies for the invasion of *P. clarkii* in China and utilization of germplasm resource for the hatchery breeding in aquaculture.

## Results

### Identification of SNP loci

Sequencing of the RAD libraries generated an average of 33.53 million reads per individual, prior to any quality filtering. After quality filtering, on average, 26.54 million (79.22%) reads with restriction site per individual were retained. Of the retained sequences, an average of 10.89 million (40.98%) aligned to the *P. clarkii* genome survey sequences (not published) with the average coverage was 26.08 ± 6.29 reads per locus. The sequences were discarded due to alternative alignments and insufficient depth of coverage (Supplementary Table S1). A total of 20,691 loci with minimum 3X coverage were retained for SNP discovery, and these RAD loci were distributed among 19,908 reference scaffolds. Of these, 19,339 (93.47%) loci were monomorphic, 1352 (6.53%) loci showed two alleles per individual and were consequently eliminated from further analyses, producing a total of 22,043 candidate SNP markers. Transition polymorphisms (Ti) outweighed transversions (Tv), accounting for 56.14% of the SNP sites in our data set, with an observed Ti: Tv ratio of 1.28 (Supplementary Fig. S1).

### Population genetic differentiation

Average observed and expected heterozygosity was 0.0047 and 0.0052, respectively. The nucleotide diversity (π) across all individuals ranged from 0.1335 to 0.1964 among 22 populations. The *F*_ST_ values between populations ranged from 0.0400 to 0.2521. Also, we calculated the *F*_ST_ based on the SNP sites (Supplementary Fig. S2A). 4099 of 22,043 loci (18.60%) gave low or negative estimates of *F*_ST_, and 8695 of the loci (39.45%) had noticeably high *F*_ST_ values (>0.15), of which 2284 even exceeded 0.25. Analysis of molecular variance (AMOVA) revealed that a significant fraction of the total variance was due to between-populations variance (12.69%, *F*_ST_ = 0.1269; *P* < 0.01) with evidence of significant genetic differentiation (*P* < 0.05); most of the variance was due to variation within populations (87.31%). Notably, the pairwise comparison of *F*_ST_ values from seven populations in the middle reaches of Yangtze River basin revealed the significant lower level of genetic differentiation between these populations than others with the average of 0.085, indicating the high level of genetic communication among the populations in the middle reaches of Yangtze River basin. The Mantel test for matrix correlation between genetic similarity and geographical distances were not significant (*r* = −0.2946, *P* = 0.9780), indicating the pattern of isolation by distance for the *P. clarkii* didn’t exist in the main distribution areas of China. Additionally, the mismatch distribution displayed a unimodal curve for *P. clarkii* (Supplementary Fig. S2B) and fit the expected distributions under sudden expansion model.

### Population genetic structure

**Figure 1:**
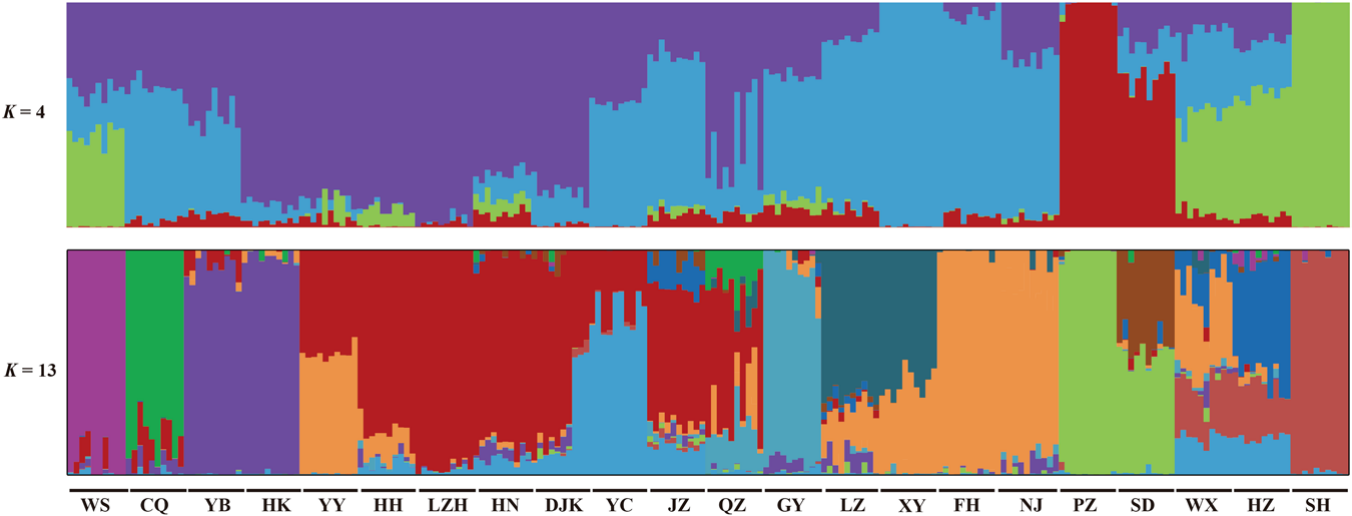
Population genetic structure of the 221 *P. clarkii* individuals. Analyses with STRUCTURE show the clustering of individuals into 4 and 13 groups. The proportion of the individual’s genome from each ancestral population is shown by the length of each colored segment.

**Figure 2:**
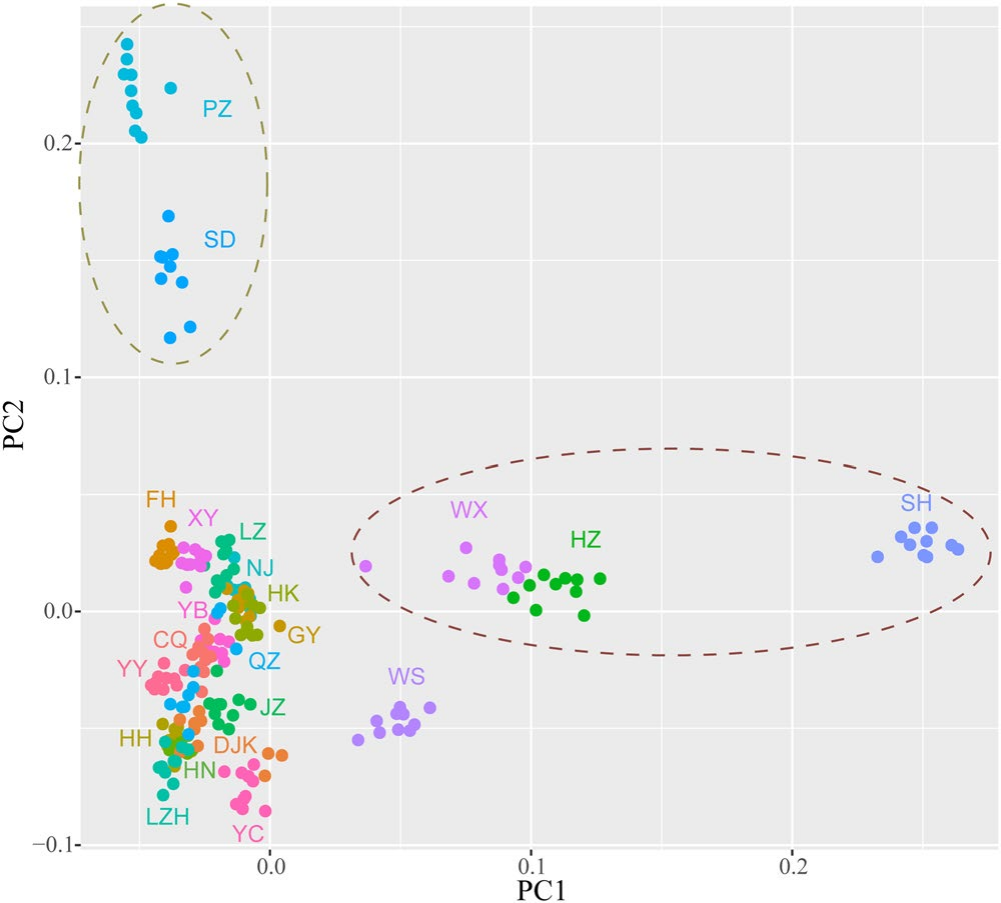
The principal components plots of 221 individuals in Yangtze River basin based on the 1st an 2nd eigenvectors. The different colors represent the different crayfish populations. The dashed circles indicate the individuals from the lower reaches of Yangtze River basin and Huai River basin, respectively.

The Bayesian analysis of population structure in STRUCTURE and calculation of Δ*K* value from the STRUCTURE output showed the model value of parameter *K* was 13, thus indicating that the uppermost hierarchical level detected by STRUCTURE was 13 distinct genetic clusters (Fig. 1). The clustering of population structure revealed that each of the populations in the upper reaches of Yangtze River basin had the specific ancestors. Three populations from the lower reaches (including WX, HZ and SH) grouped into one cluster, and showed the similar population structure. Two populations from Huai River basin (SD and PZ) exhibited the different ancestors with other populations. With the *K* equal to four, we found the obvious division between the WX, HZ and SH populations, two populations from Huai River basin and the populations from the upper reaches of the Yangtze River above Nanjing City, which was consistent with the population’s geographic distribution. The WS population shared the ancestry sequences slightly with the WX, HZ and SH populations, indicating high level of gene flow between these populations. The principal components analysis (PCA) also recovered these groupings (Fig. 2), with the first and second eigenvectors separated the populations into three subgroups, i.e., two populations of Huai River basin including PZ and SD, three populations of lower reaches of Yangtze River including WX, SH and HZ, the other populations in the upper reaches of the Yangtze River above Nanjing City. Here, we defined these three subgroups as three regions, including Huai River region, lower reaches region and upper and middle reaches (UM) region, according to the population structure and geographic location of these populations.

**Figure 3:**
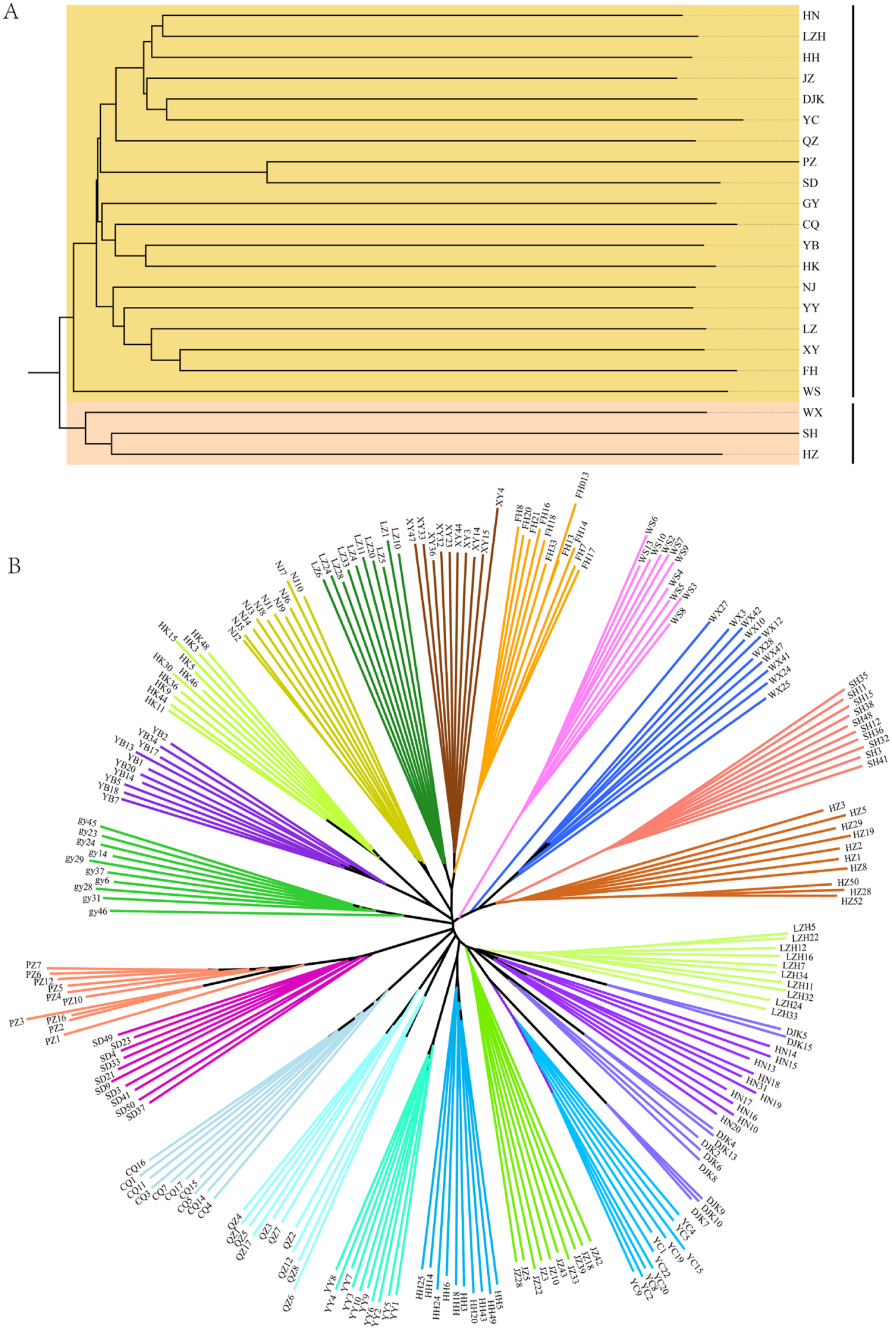
Neighbor-joining trees of the 22 crayfish populations (**A**) and the 211 individuals (**B**). The different colors in (**B**) indicate the individuals from different populations.

The neighbor-joining (NJ) tree of these crayfish populations (Fig. 3A) features two main clusters, with HZ, SH and WX populations grouping together and the other populations forming the other cluster. Notably, the individuals (e.g., CQ, YB and WS) from upper reaches irregularly clustered with the individuals from middle reaches of Yangtze River basin. For instance, the YB and CQ populations were clustered with HK population. It could be inferred that the populations from middle reaches invaded into the upper reaches of Yangtze River basin with high level of human-mediated dispersal in the past few decades. The NJ tree of the 221 individuals (Fig. 3B) revealed that the individuals from same location grouped into one population-specific cluster except the individuals in DJK population, which indicated the high level of genetic diversity in DJK population. The branch length of the NJ tree exhibited the length of individual or population-specific branches account for the most proportions, inferring that the major genetic variations of crayfish derive from the among-individuals within population. These findings also coincided with the results of analysis of molecular variance.

### Gene flow of *P. clarkii* populations

**Figure 4:**
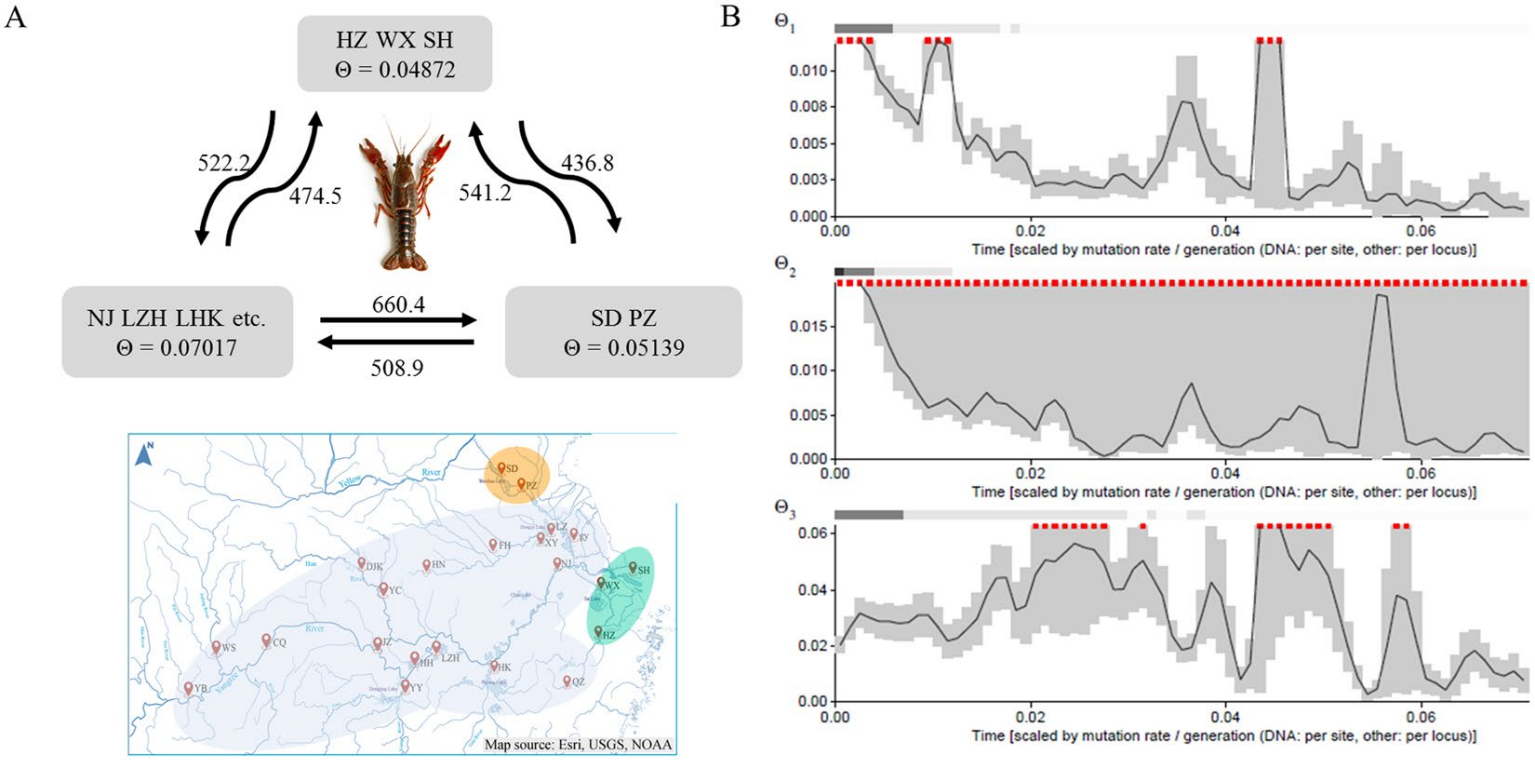
Estimates of the migration (*M* and *θ*) (**A**) and skyline plots among three geographic regions of crayfish (**B**). *Θ* represents the mutation-scaled population size, and the value *M* in (**A**) indicates the mutation-scaled migration rate. *Θ*1, *θ*2, *θ*3 represents the population sizes of Huai River, lower reaches and UM regions of Yangtze River basin, respectively. The map in (**A**) was generated by ArcGIS 10.2.

The population structure and NJ tree revealed that these 22 populations grouped into three clusters, which coincided with the geographic distribution. Here, we constructed a gene flow hypothesis that persistent asymmetrical gene flow occurred among these three regions. To investigate the migration among these three regions, the population sizes and migration rates of each group were calculated using Migrate-n program. Three geographical regions were analyzed, and estimates of effective population size (*θ*) were consistently low and ranged from 0.0487 for lower reaches region (SH, HZ and WX) to 0.0702 for the UM region (Fig. 4A). Estimates of migration rate between three regions were bi-directional and relatively low, which ranged from 436.8 to 660.4. The highest *M* value was found for individuals from UM region to Huai River region, while the lowest *M* value was for individuals from the lower reaches region to Huai River region. Significant asymmetrical migration rates between UM region and Huai River region were discovered by non-overlapping 95% confidence intervals of each estimate. Skyline plots revealed that the population sizes of UM region and lower reaches region increased prior to Huai River region, and the population sizes of UM and Huai River region were higher than the lower reaches region (Fig. 4B).

### Phenotypic divergence of carapace width in *P. clarkii*

**Figure 5:**
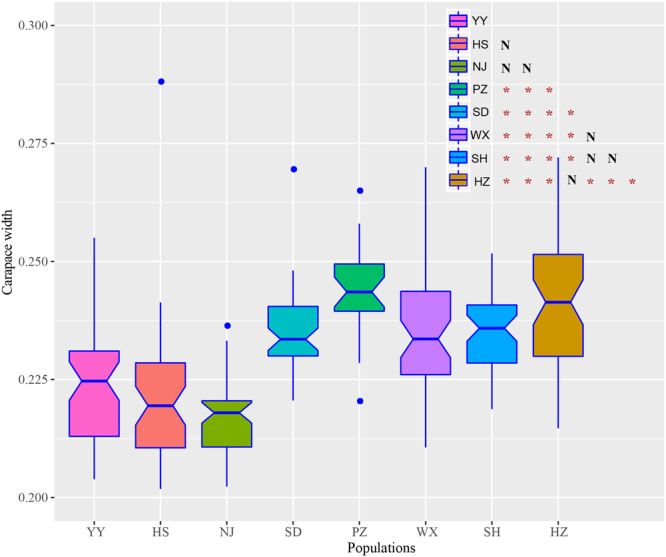
The morphological divergence of carapace width in the eight populations. The symbols below the diagonal in the top right corner represent the significance level in ANOVA. N indicates *P* > 0.05; asterisk indicates *P* < 0.05.

Since the level of genetic differentiation is relatively low between populations and is higher within populations. Investigation of morphological characters between different populations of *P. clarkii* could contribute to understanding the rapid evolution of phenotypic traits within populations of the crayfish to some extent. A total of 378 female individuals from eight locations were chosen for morphological investigations. Significant positive relationships existed between body weight and the other six traits (i.e., body length, carapace length, carapace width, abdominal segment length, abdominal segment width, and first abdominal segment length) in *P. clarkii* (*P* < 0.05). Path analysis showed that carapace length (*L*_C_), carapace width (*W*_C_) and abdominal segment length (*L*_A_) had strong positive direct effects (Table 1), indicating these three characters, especially carapace length (*L*_C_) and carapace width (*W*_C_), are indicative of determining the body weight of *P. clarkii*. In total, 90.4% of the variation in weight could be explained by the variation of two independent variables. The unexplained variation, 9.6% of the total, may be due to variation in the other components under consideration. To investigate the phenotypic divergence of the individuals from different populations, we compared the five external characters among the populations, which were standardized by total length (*L*_T_). The results showed that carapace widths of *P. clarkii* were differed among these populations (Fig. 5). Three populations (HS, YY and NJ) in the UM region exhibited a significant lower carapace width/total length ratio than other populations in lower reaches region and Huai River region (Wilcoxon rank sum test, *P* < 2.2 × 10^−16^). The lowest value of carapace width/total length ratio was observed in NJ population, where *P. clarkii* was firstly introduced into China. These populations were divided into three groups based on the geographic distribution. Further, the phenotypic divergence of carapace width between the populations in the lower reaches (WX, SH and HZ) and Huai River region (SD and PZ) was not significant (*P* = 0.38).

**Table 1:** Path coefficients for measured characters in *P. clarkii*.

Variables	Correlation coefficients	Path coefficients	Indirect effect		
			*L*_C_	*W*_C_	*L*_FA_
*L*_C_	0.948*	0.758*	—	0.137	0.054
*W*_C_	0.895*	0.149*	0.695	—	0.051
*L*_A_	0.842*	0.062*	0.657	0.122	—
*L*T	0.916*	0.064			
*L*_FA_	0.805*	0.006			
*W*_A_	0.802*	−0.016			

Total length (*L*_T_), carapace length (*L*_C_), abdominal segment length (*L*_A_), first abdominal segment length (*L*_FA_), abdominal segment width (*W*_A_) and carapace width (*W*_C_); ^∗^Significant at the 0.05 probability level.

## Discussion

*P. clarkii*, one of the most famous invasive species in China, dramatically became an important cultured species in China. Since it invaded into China in 1920s, it has widely dispersed in Yangtze River basin and Huai River basin. Barbaresi *et al*.^3^ proposed that the wide dispersal of *P. clarkii* throughout the world is attributed to human activities. It is evident that aquaculture and aquatic product transportation promote the spread of this invasive species in China. Also, the dispersal capabilities (behavioral flexibility) of *P. clarkii* contribute to the rapid and widespread diffusion. Therefore, population genetic structure, genetic diversity and evolutionary history, and phenotypic divergence of this alien species deserve a special mention in response to challenges in biodiversity conservation and invasion control.

### Characterization of population genetic structure

It is generally known that crustaceans were considered to be problematic for karyological and cytogenetic studies, as the majority of species have relatively high diploid chromosome numbers (>150) and relatively small chromosomes^23^, and *P. clarkii* is no exception^24^. Therefore, the genome assembly of *P. clarkii* becomes much more difficult even though the NGS technology rapidly developed in the past few years. As an efficient and flexible method for genome-wide genotyping, 2b-RAD technique^17^ provides an excellent fractional representation of the targeted genome and has been widely applied in the previous studies^25–27^. In this study, to reveal the characteristic of genetic structure of *P. clarkii*, a total of 22,043 SNP sites were identified from the *P. clarkii* genome based on 2b-RAD technique, which were adequate for the study of population genetics.

The high level of genetic diversity in *P. clarkii* populations was detected in this study, which is consistent with the previous studies^12^ and could contribute to invasion success^28^. Meanwhile, the high level of variance within populations rather than among populations was revealed in this study, which might help to the invasion success of *P. clarkii*^11,12^, and also it appears to be an typical genetic feature of invasive species after its successful invasion, which is also reported in other invasive species^29–31^. Meanwhile, the high *F*_ST_ values revealed less genetic exchange between most *P. clarkii* populations, which may be related to its migrational ability in the wild and high fecundity. In addition, the low rate of migration between natural populations, which is affected by the human activities (extensive fishing of this economic species), to some extent reduced the level of genetic exchange between the populations in China. Furthermore, other methods should be adopted to further confirm this conclusion in the future studies, such as investigation with slow evolving sites or haplotype distribution. Additionally, no pattern of isolation by distance (IBD) detected in the *P. clarkii* populations to some extent further verified that human-mediated jump dispersal was the main dispersal pattern for *P. clarkii*.

The populations from upper reaches of Yangtze River basin exhibited similar population structure with the populations from middle reaches. Li *et al*.^11^ proposed that the *P. clarkii* was most likely originally introduced into China from Japan, and then expanded its range primarily into the middle and lower reaches. Here, we infer that the populations in upper reaches may originate from the populations in middle reaches of Yangtze River basin. Notably, two populations (SD, PZ) from Huai River region and three populations (SH, WX, HZ) from lower reaches region obviously separated from UM region, which coincided with the phenotypic divergence of carapace width. These three regions revealed asymmetrical migration and low migration rates. Meanwhile, the mismatch distribution was adopted to investigate the demographic history of *P. clarkii*, and the typical unimodal distribution indicated that populations had undergone demographic expansions. To estimating population expansion through time, Bayesian skyline plots revealed that the population sizes of three regions were fluctuant and experienced one or two period of rapid increase. Interestingly, the population size (*θ*) of UM region remained at high levels in the longtime, which experienced two periods of rapid increase. These findings confirmed our hypothesis that persistent asymmetrical gene flow occurred among these three regions, and UM region was the main distribution area. The population sizes of lower reaches and Huai River regions were increasing, which were consistent with the conclusion reported in Li *et al*.^11,12^. In summary, this successfully invasive species that was mainly influenced by human-mediated dispersal revealed some characteristics of genetic landscape including asymmetrical migration, population size rapid increasing, high genetic differentiation within population, etc.

As the characteristics for an successfully invasive species mentioned above, the notion of fast evolving DNA markers reaching saturation levels of genetic distance between populations and maximum genetic diversity (MGD) theory^32,33^ might account for the phenomenon that the low genetic differentiation among populations and high differentiation within population were detected in this study. Unfortunately, it was difficult to carry out the related analysis in this species because it seemed impossible to identify the slow evolving sites based on the limited SNPs information in this study. The bottleneck was that the reads outputted from 2b-RAD technique was only 33 bp in length. A comprehensive conclusion could not be given for the results mentioned above due to the limitations of the SNPs with limited information used in this study. The identification of more informative SNPs in *P. clarkii* must be carried out using other better methods (e.g., genome resequencing, amplicon sequencing) in the future studies. In addition, the crayfish genomic information is still very limited although we had sequenced the genomic sequences. The SNPs related with phenotypic divergence could not be identified even though the morphological characters were investigated in the subsequent section. Thus we could only exhibit the trend of divergence of morphological traits for *P. clarkii* as a whole.

### Phenotypic divergence of *P. clarkii* after its rapid invasion in China

Generally, invasive species show higher phenotypic plasticity than native species^34^, and high level of phenotypic plasticity should enable a colonizing species to cope with, and become established under new environments. Many studies have focused on the phenotypic differences between populations in invasive plants^35–38^ (e.g., *Impatiens glandulifera*, *Bromus tectorum*, *Agrostis capillaris*, *Eschscholzia californica*), and fewer studies have proposed the phenotypic divergence of invasive animals^39,40^. As a famous invasive species, *P. clarkii* provide a desirable opportunity to investigate rapid adaptation of its phenotype. Given that the genome complexity of *P. clarkii*, phenotypic divergence is a better breach for predicting future invasion scenarios and understanding how populations can evolve rapidly in response to novel and changing environments. In the present study, the morphological differentiation of carapace width in *P. clarkii* was observed after invaded into China, and the lowest value of carapace width was observed in NJ population. Since it was firstly introduced into Nanjing City and invaded other areas^10^, the hypothesis that the carapace has been gradually widening for the rapid evolution of *P. clarkii* after its invasion was supported by the comprehensive comparisons of morphological characters. Therefore, it could be inferred that rapid evolution or selection affects the phenotypic change of the different populations in *P. clarkii*. Path analysis revealed that higher carapace width indicates the higher weight of individuals in *P. clarkii*, which are exclusively preferred in aquaculture at present.

### Management implications and scientific utilization of *P. clarkii*

*P. clarkii* plays two roles in China, a famous invasive species and a popular aquaculture species. The middle reaches of Yangtze River dominates the crayfish industry of China in both aquaculture and wild capture fisheries, where the industry contributes well in excess of 56.4 billion RMB annually in 2016 (www.cnfm.gov.cn/). Although the burrowing activities of *P. clarkii* can lead to damage to water courses and to crops, particularly rice, and its feeding can disrupt native ecosystems^41^, Chinese farmers are adept at avoiding the ecological damage (i.e., building blocking net) and utilization of its biological characteristics (i.e., tolerating relatively low dissolved oxygen concentrations and high temperatures). For example, the rice-fish culture system, a complex ecological aquaculture mode, is widely developed in southern China^42^. *P. clarkii* is better adapted to growing in a rice field, and the concurrent culture of rice and crayfish makes good use of land, resources, equipment, and infrastructure already being used for rice production. Also, the wild capture of *P. clarkii* is still in a certain proportion in Chinese crayfish industry presently, while the wild crayfish’s burrows result in dam damages and a huge loss of irrigation water, causing significant economic loss^43^.

The high level of genetic diversity in *P. clarkii* populations and low level of genetic exchange between most crayfish populations were observed in our study. High level of genetic diversity promotes availability of natural variations for use in breeding germplasm. The *P. clarkii* populations with high level of genetic variation might be used as a source for selection of desirable germplasm and development of new varieties. In addition, the carapace of *P. clarkii* has been widening since it was firstly introduced into China in 1920s. Path analysis revealed that the characters in carapace play major role in body weight of *P. clarkii*. It provides novel insights into future selective breeding of *P. clarkii*, for improving the economic value of crayfish culture. The results of the present study, together with those of previous population genetic studies in *P. clarkii*^11,12^ investigated the comprehensive genetic information of *P. clarkii* populations in China, could provide a significant guidance into utilization of the valuable genetic resources and more efficient management of its invasion.

Overall, this study provided some suggestive clues for comprehensively revealing the impact of human-mediated dispersal on the genetic structure and diversity of *P. clarkii* populations, and uncovered the population genetic landscape of this famous aquatic animal as well as the phenotypic differentiation between different areas in China that could provide us some fresh ideas to conduct selective breeding program in future. However, imperfectly, this study has two weaknesses that should be considered when interpreting these results. First, we used only a small number of populations from the Huai River region to draw conclusions as a whole. A larger population size would be an important way to make more powerful conclusions about migration rate, population structure, and genetic diversity. Also, the gene flow from unsampled populations in Huai River region could be influencing the poor estimates because Migrate-n assumes that all populations exchanging migrants are sampled. Second, the sequencing method used in this study had few limitations that the SNPs identified based on this method without high-quality reference genome were not informative due to the generation of short reads (33 bp in length). However, such an ideal assumption cannot meet for most studies, yet this method has been widely used to infer migration rates in natural populations. Given the above mentioned, our future work will collect more samples from more areas especially the Huai River region if possible, and focus on evaluating the evolving rates of genomic variants that contribute to understanding the genetic diversity and phylogeny of *P. clarkii* and the potential connectivity between environmental conditions and genetic variations.

## Methods

### Samples collection

**Figure 6:**
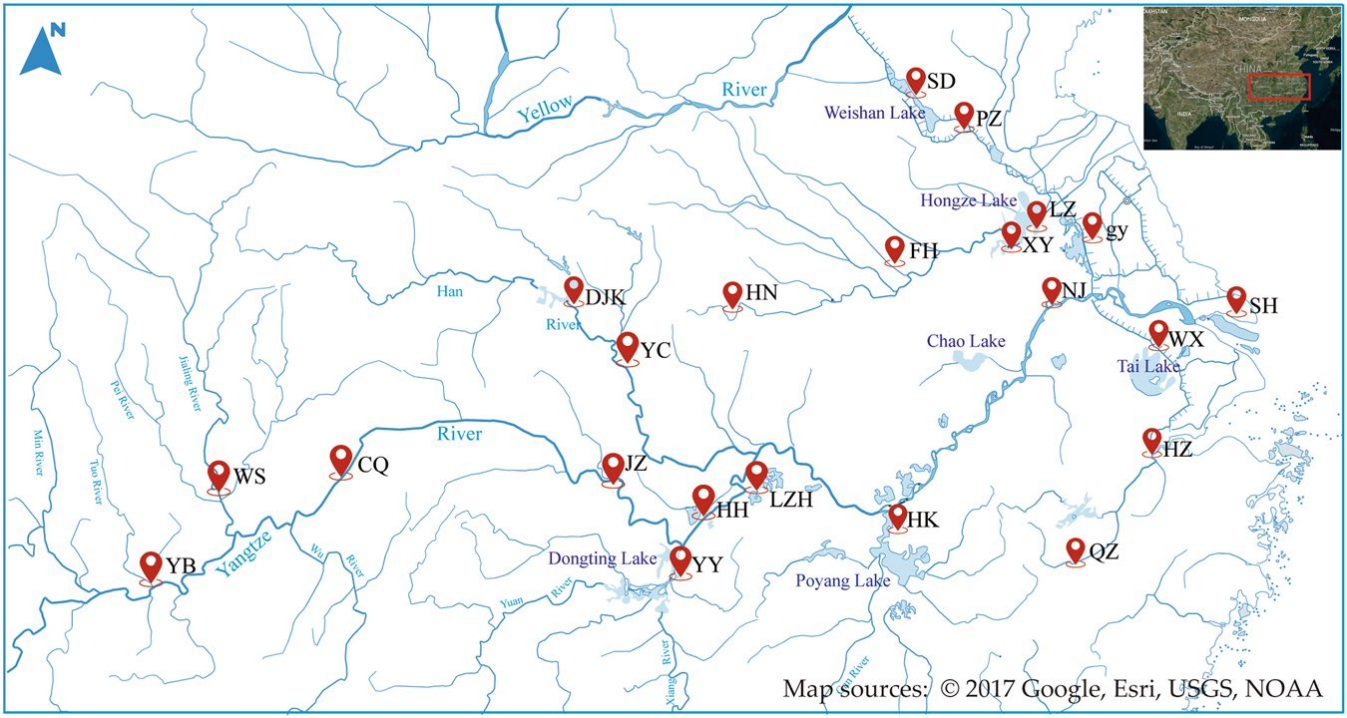
Geographic locations of 22 *P. clarkii* populations collected for 2b-RAD in China. The map was generated using ArcGIS 10.2.

To explore the genetic dynamics and genetic structure of *P. clarkii*, a total of 22 natural populations, which selected from the major cultivation areas and river basins where *P. clarkii* distributed in China, were collected (Fig. 6; Supplementary Table S2). Ten specimens of each location were sampled, and the muscle tissue was preserved in 95% ethanol and stored at 4 °C for later DNA extraction. Additionally, seven populations from 22 populations mentioned above and one population from Huangshan, Anhui province were selected to evaluate the phenotypic differentiation (Supplementary Table S2; Supplementary Fig. S3).

### SNP identification

Total DNA was extracted from muscle tissue using the ammonium acetate method^44^. Extracted DNA was dissolved in DNAase-free water and concentration was determined using NanoDrop 2000 spectrophotometer (Thermo Fisher Scientific, Waltham, USA). DNA integrity was assessed by 1.0% agarose gel electrophoresis. The 2b-RAD libraries were constructed for each individual following the methods from Wang *et al*.^17^ Different from original 2b-RAD technique, our procedure produced single-tag constructs using modified adaptors and biotin-labeled primers, and then digested by the *Bsa* XI enzyme to generate distinct cohesive ends and then ligated in a predefined order to produce five concatenated tags for Illumina paired-end sequencing. Meanwhile, a draft genome of *P. clarkii* was assembled with 102.73 Gb clean data, and the maximum contig was 83,480 bp in length and the contig N50 value of 1,138 bp (unpublished). This genome was adopted as a reference which was used in the subsequent analysis. The restriction enzyme recognition sites of *Bsa* XI were detected on the genome and the recognition sites number was 99,273 in the scaffolds.

To assess the robustness of the method and subsequent data analyses, FH11 library was replicated (Technical Replicates, TRs). The starting DNA was digested with the *Bsa* XI restriction enzyme and ligated to the two library-specific adaptors. To reduce marker density, the adaptors with fully degenerate 5′-NNC-3′ overhangs were chosen. For estimating *Bsa* XI sites coverage, simulant detections of the *Bsa* XI sites in *P. clarkii* reference genome were carried out. PE300 sequencing of 221 libraries was performed on Hiseq X10 platform (Illumina, Inc. USA).

Demultiplexed reads were returned by the sequencing facility in FastQ format and their quality was checked by FastQC (www.bioinformatics.babraham.ac.uk/projects/fastqc/). The Perl scripts from Wang *et al*.^17^ were run for quality filtering and adaptors trimming of the reads. Assembly of the PE reads and extraction the assembled reads containing five tags was performed on PEAR script. The processed five-tag dataset from PEAR output was divided into single-tag data sets using a Perl script, and then the single tags with restriction site were extracted. These tags of each individual were mapped to the reference genome using SOAP2 program (http://soap.genomics.org.cn/) with the parameter −M 4, −v 2 and −r 0 allowing four mismatches. The sequences that aligned with only one location were regarded as unique tags and the expected potential markers. The aligned reads were performed SNP calling using RADtyping^45^ with default Posterior probability is calculated for two possible genotypes (homozygote and heterozygote) at a given locus using a maximum likelihood approach. To look for the relationship between sequencing data size and *Bsa* XI sites coverage, a correlation test was performed based on two repeated samples. To improve the accuracy of SNP genotyping, the loci that were present in all samples in at least 80% of the individuals from each sample, with at least three RAD tags per allele at each locus (3X coverage per allele) were included. To avoid linkage bias for the SNP calling, only the loci containing 1~2 SNP and minor allele frequency (MAF) >0.05 were retained in the final analysis. The SNP dataset is publicly available at the website: https://doi.org/10.6084/m9.figshare.5537584.v1.

### Genetic diversity and population genetic analyses

Pairwise F-statistics (*F*_ST_) per pair of populations were calculated using Arlequin suite version 3.5^46^ to identify genetically differentiated localities. The potential number of genetic clusters and the membership of each individual were estimated using STRUCTURE Ver. 2.3.4^47^. The software uses Markov chain Monte Carlo (MCMC) simulations to estimate those parameters, with the number of clusters to be tested (*K*) specified by the user. The MCMC simulation was run for 300,000 repetitions, after a burnin period of 100,000. For each value of *K* (number of potential ancestral populations), the genetic ancestry of each individual was estimated based on the admixture model without any prior population assignment; estimations were obtained from the 300,000 iterations that followed a burn-in period of 100,000 iterations. The correlation between genetic distance and geographic distance was assessed using IBDWS version 3.21^48^. Additionally, the distribution of genetic variation was analyzed by AMOVA analysis using the Arlequin suite version 3.5. Principal components analysis (PCA) was performed using GCTA software^49^, and the scatter plot of the first and second components was done using R (https://www.r-project.org/). We then used these average pairwise *F*_ST_ values to cluster populations by a neighbor-joining method implemented in the MEGA 6.0 program^50^. Patterns of migration and past migration rates between populations were determined with MIGRATE-N program following the procedure described by Beerli and Palczewski^51^. The tested connectivity model was chosen based on the hydrography of the region and the previous genetic structure analysis. Full migration model with all migration paths open (asymmetric gene flow allowed) was evaluated in the analysis.

### Morphological differentiation in *P. clarkii*

To investigate the divergence of morphological traits in *P. clarkii* which evolved over a timescale of about 80 years in China, eight natural populations (see Supplementary Fig. S3) were selected to evaluate the phenotypic patterns. More than 30 individuals of each population were measured using an electronic digital caliper. Seven morphological characters, including Weight (*W*), total length (*L*T), carapace length (*L*_C_), abdominal segment length (*L*_A_), first abdominal segment length (*L*_FA_), abdominal segment width (*W*_A_) and carapace width (*W*_C_), were investigated. Simple correlation and stepwise multiple regression analysis were performed using SPSS program (IBM, USA). The relative importance of direct and indirect effects of measured traits on weight was determined by path analysis for the morphological data. In the path analysis, weight was the dependent variable and the six characters (mentioned above) were considered as independent variables. Given the variables of the individual size, total length was used to standardize the size effect in the statistical analysis. The statistics were executed in R (https://www.r-project.org/) using the package coin^52^. Significance was assumed if *P* < 0.05.

### Data Availability

Raw sequence data were deposited into the NCBI Short Read Archive (SRA) with the accession number SRP135662.

## Electronic supplementary material

Dataset 1
