# Supplementary material for: Characterization of Population Genetic Structure of red swamp crayfish, Procambarus clarkii, in China
Source: Sci Rep. 2018 Apr 3;8:5586. doi: 10.1038/s41598-018-23986-z (PMC5883011; doi:10.1038/s41598-018-23986-z)
Supplement: Supplementary file 1 — Dataset 1 [file 41598_2018_23986_MOESM1_ESM.zip › Supplementary dataset.pdf]

Characterization of Population Genetic Structure of red swamp crayfish, *Procambarus clarkii*, in China

Shaokui Yi[1,2], Yanhe Li[1,*], Linlin Shi[1], Long Zhang[1], Qingbin Li[1] & Jing Chen[3]

[1] College of fisheries, Key Lab of Agricultural Animal Genetics, Breeding and Reproduction of Ministry of Education, Huazhong Agricultural University, Wuhan, 430070, P. R. China

[2] Fish Genetics and Breeding Laboratory, The Ohio State University South Centers, Piketon 45661, Ohio, United States of America

[3] Institute of Fisheries, Anhui Academy of Agricultural Sciences, Hefei 230031, P. R. China

Figure S1

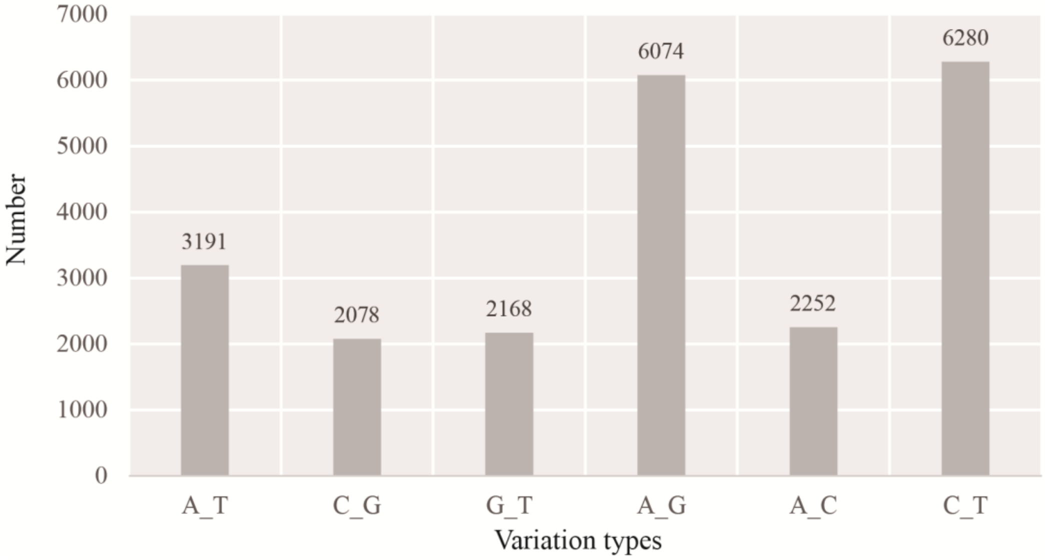

Figure S1 Transitions and transversions occurring within a set of 22,043 *P. clarkii* SNPs.

Figure S2

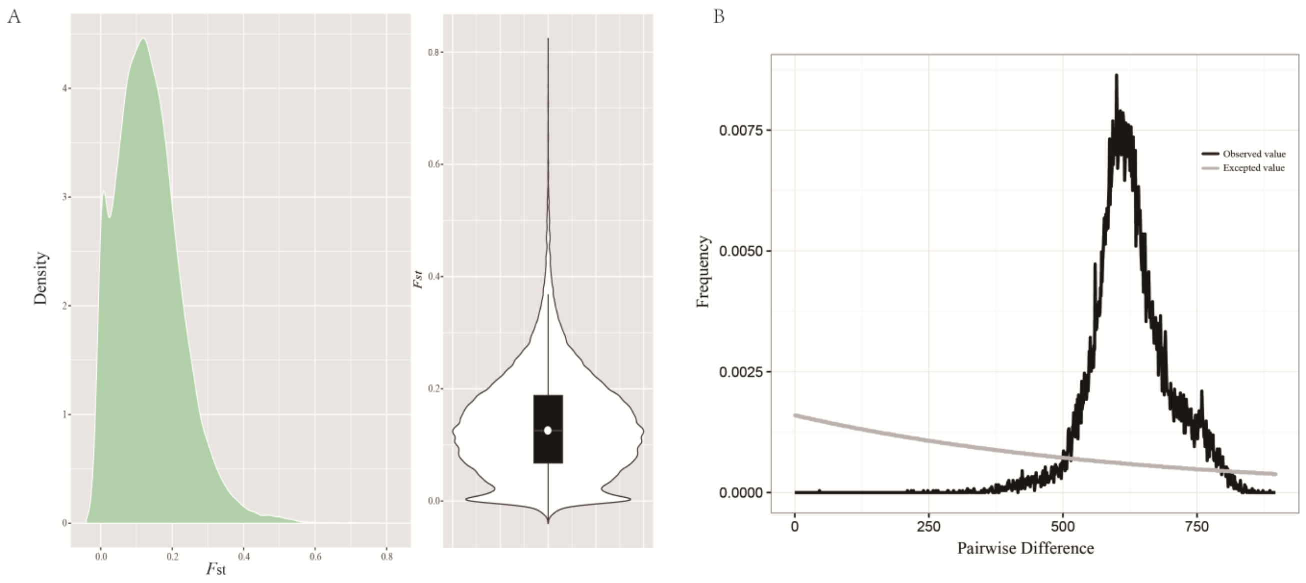

Figure S2 The distribution of F_{ST} values (A) and mismatch analysis (B) based on the *P. clarkii* SNPs.

Figure S3

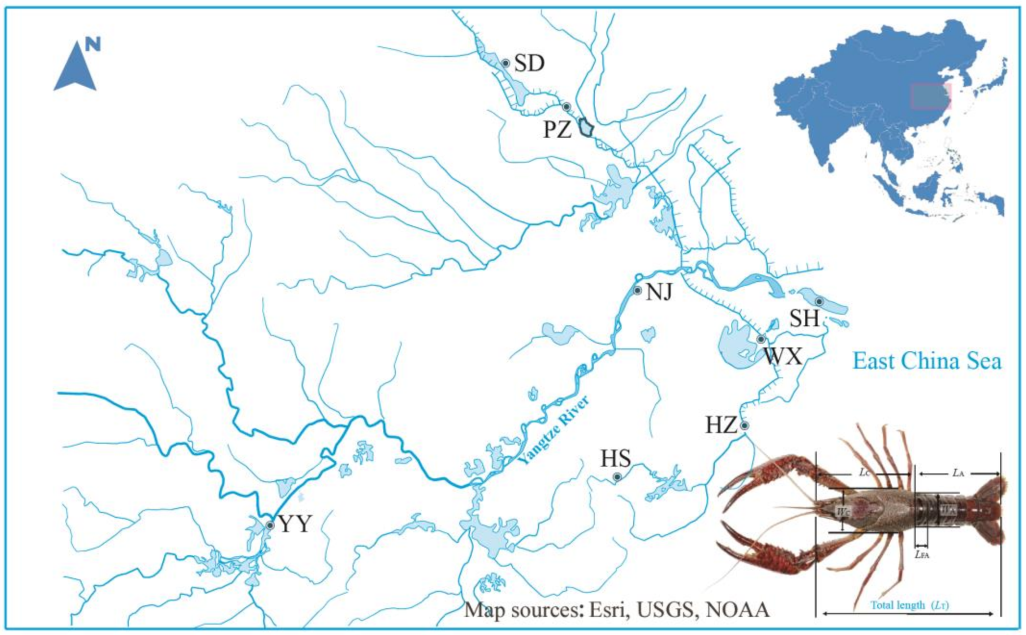

Figure S3 The geographic locations of eight populations collected for morphological analysis in this study. This Figure was generated by ArcGIS 10.2 and Adobe Illustrator CS5.1 software.

Table S1 Sequencing information of 221 individuals in 22 populations

Sample	Clean data	Enzyme	Percentage	Tag number	Depth	Mapping rate
PZ1	34,333,423	25,699,409	74.85%	459,702	20.94	37.46%
PZ2	34,333,423	25,708,471	74.88%	452,789	21.95	38.67%
PZ3	34,333,423	21,343,228	62.16%	274,392	33.67	43.28%
PZ4	34,333,423	24,159,869	70.37%	307,233	32.36	41.15%
PZ5	31,979,114	28,121,363	87.94%	445,127	24.77	39.20%
PZ6	31,979,114	24,927,849	77.95%	455,842	22.72	41.54%
PZ7	31,979,114	24,514,013	76.66%	306,074	36.14	45.13%
PZ10	31,979,114	19,963,395	62.43%	295,511	27.70	41.00%
PZ12	29,728,292	19,573,362	65.84%	425,219	17.28	37.53%
PZ16	29,728,292	21,188,360	71.27%	429,105	19.19	38.87%
HH3	29,728,292	25,180,046	84.70%	321,076	35.60	45.39%
HH5	29,728,292	23,845,271	80.21%	320,216	31.61	42.45%
HH6	32,579,510	27,507,643	84.43%	489,725	21.73	38.68%
HH14	32,579,510	26,751,314	82.11%	479,097	23.27	41.67%
HH18	32,579,510	27,160,625	83.37%	323,784	37.74	44.99%
HH20	32,579,510	24,132,635	74.07%	324,236	31.54	42.37%
HH24	31,169,305	25,724,105	82.53%	487,831	20.76	39.36%
HH25	31,169,305	22,850,880	73.31%	476,142	20.38	42.46%
HH43	31,169,305	23,302,331	74.76%	313,309	35.27	47.42%
HH49	31,169,305	25,442,880	81.63%	321,848	33.95	42.94%
XY47	42,382,243	36,087,067	85.15%	433,993	34.51	41.50%
FH7	33,259,472	27,526,140	82.76%	486,972	22.92	40.55%
FH8	33,259,472	27,837,319	83.70%	478,672	23.45	40.33%
FH13	33,259,472	23,249,758	69.90%	271,684	37.76	44.12%
FH14	33,259,472	26,590,847	79.95%	317,579	34.32	40.99%
FH16	34,621,853	25,549,103	73.79%	488,152	20.35	38.88%
FH17	34,621,853	26,145,011	75.52%	490,678	21.72	40.76%
FH18	34,621,853	27,396,574	79.13%	327,175	37.58	44.88%
FH20	34,621,853	25,572,042	73.86%	327,305	32.20	41.21%
FH21	30,978,280	23,265,901	75.10%	480,439	18.88	38.99%
FH33	30,978,280	22,236,558	71.78%	463,977	18.79	39.21%
WS2	30,978,280	22,600,488	72.96%	305,183	33.01	44.58%
WS3	30,978,280	19,406,459	62.65%	294,912	27.81	42.26%
WS4	31,868,091	23,395,934	73.41%	477,471	19.86	40.53%
WS5	31,868,091	24,751,665	77.67%	476,751	20.85	40.16%
WS6	31,868,091	22,278,107	69.91%	290,502	33.81	44.08%
WS7	31,868,091	23,867,404	74.89%	313,589	33.18	43.60%
WS8	36,188,506	26,060,715	72.01%	497,920	20.62	39.39%
WS9	36,188,506	28,595,339	79.02%	488,199	23.61	40.31%
WS10	36,188,506	26,655,166	73.66%	315,105	36.87	43.58%
WS13	36,188,506	26,232,984	72.49%	326,354	33.83	42.08%
YB1	33,357,421	22,085,943	66.21%	464,026	18.86	39.63%

Continue

YB2	33,357,421	27,494,808	82.42%	482,602	23.07	40.49%
YB5	33,357,421	22,695,980	68.04%	313,091	31.63	43.63%
YB7	33,357,421	23,424,570	70.22%	326,585	30.28	42.21%
YB13	30,904,184	25,292,155	81.84%	478,205	21.90	41.41%
YB14	30,904,184	19,910,752	64.43%	448,604	17.82	40.15%
YB17	30,904,184	20,920,325	67.69%	308,314	31.49	46.41%
YB18	30,904,184	18,753,166	60.68%	294,418	27.27	42.82%
YB20	32,580,444	23,978,737	73.60%	471,481	20.37	40.06%
YB34	32,580,444	26,481,628	81.28%	471,167	23.71	42.19%
CQ1	32,580,444	19,575,605	60.08%	300,720	29.10	44.71%
CQ3	32,580,444	19,259,307	59.11%	292,002	26.88	40.75%
CQ4	33,030,282	20,924,453	63.35%	431,042	18.33	37.76%
CQ5	33,030,282	22,899,486	69.33%	471,369	19.55	40.24%
CQ7	33,030,282	24,148,490	73.11%	322,110	33.64	44.88%
CQ11	33,030,282	26,508,236	80.25%	323,711	34.50	42.13%
CQ14	30,726,619	23,642,260	76.94%	468,465	19.68	38.99%
CQ15	30,726,619	24,706,233	80.41%	476,440	20.76	40.03%
CQ16	30,726,619	22,917,157	74.58%	320,832	31.95	44.72%
CQ17	30,726,619	20,628,956	67.14%	319,648	26.94	41.75%
HN10	38,929,287	27,016,772	69.40%	495,537	21.33	39.13%
HN13	38,929,287	26,861,443	69.00%	482,143	22.96	41.21%
HN14	38,929,287	23,429,557	60.18%	318,824	32.11	43.69%
HN15	38,929,287	31,924,090	82.01%	349,277	38.07	41.65%
HN16	35,727,526	19,386,776	54.26%	468,134	15.73	37.99%
HN17	35,727,526	26,642,723	74.57%	489,208	21.76	39.95%
HN18	35,727,526	26,726,540	74.81%	441,855	26.39	43.63%
HN19	35,727,526	26,629,254	74.53%	452,217	23.81	40.43%
HN20	32,693,981	18,102,049	55.37%	459,317	15.12	38.36%
HN31	32,693,981	20,676,347	63.24%	469,976	16.89	38.38%
DJK2	32,693,981	20,420,149	62.46%	421,607	20.78	42.90%
DJK4	32,693,981	25,766,009	78.81%	441,320	24.28	41.58%
DJK5	33,378,722	21,862,138	65.50%	474,194	17.92	38.87%
DJK6	33,378,722	23,523,390	70.47%	476,340	19.47	39.42%
DJK7	33,378,722	20,421,133	61.18%	413,192	21.02	42.52%
DJK8	33,378,722	23,820,048	71.36%	444,102	21.30	39.72%
DJK9	40,425,646	26,889,798	66.52%	501,419	20.68	38.56%
DJK10	40,425,646	31,307,413	77.44%	504,586	24.54	39.56%
DJK13	40,425,646	30,064,336	74.37%	464,542	27.96	43.20%
DJK15	40,425,646	27,957,454	69.16%	460,393	24.50	40.35%
YC1	32,041,951	24,505,975	76.48%	473,882	20.36	39.38%
YC2	32,041,951	26,896,058	83.94%	472,880	22.18	39.00%
YC4	32,041,951	27,349,356	85.35%	425,853	27.15	42.27%
YC5	32,041,951	26,818,975	83.70%	429,321	24.95	39.94%

Continue

YC8	31,791,612	23,587,548	74.19%	466,568	19.55	38.67%
YC9	31,791,612	26,794,822	84.28%	478,698	22.40	40.02%
YC15	31,791,612	26,554,124	83.53%	432,143	26.14	42.54%
YC19	31,791,612	25,647,971	80.68%	455,334	22.78	40.45%
YC20	33,661,069	22,342,092	66.37%	471,794	18.77	39.63%
YC22	33,661,069	28,337,540	84.18%	473,899	23.92	40.00%
JZ3	33,661,069	26,353,394	78.29%	447,510	24.67	41.90%
JZ5	33,661,069	28,290,389	84.04%	420,021	23.26	34.53%
JZ10	37,807,558	27,391,202	72.45%	497,497	20.33	36.93%
JZ18	37,807,558	28,210,440	74.62%	489,558	20.21	35.07%
JZ22	37,807,558	27,097,013	71.67%	450,371	25.67	42.67%
JZ28	37,807,558	28,924,510	76.50%	433,153	21.70	32.50%
JZ33	27,673,347	20,930,341	75.63%	450,665	17.95	38.65%
JZ39	27,673,347	22,779,434	82.32%	462,336	18.81	38.18%
JZ42	27,673,347	20,829,272	75.27%	425,299	20.30	41.45%
JZ43	27,673,347	22,147,123	80.03%	428,822	20.32	39.35%
LZH5	39,926,067	27,644,292	69.24%	496,222	22.75	40.84%
LZH7	39,926,067	32,570,124	81.58%	504,819	24.99	38.73%
LZH11	39,926,067	33,414,061	83.69%	450,595	31.53	42.52%
LZH12	39,926,067	30,307,561	75.91%	456,129	27.21	40.95%
LZH16	37,435,308	23,722,353	63.37%	485,050	18.66	38.16%
LZH22	37,435,308	30,274,686	80.87%	495,398	24.55	40.17%
LZH24	37,435,308	26,957,258	72.01%	460,339	24.95	42.60%
LZH32	37,435,308	28,458,643	76.02%	451,865	25.42	40.36%
LZH33	34,931,138	27,100,548	77.58%	487,574	21.69	39.02%
LZH34	34,931,138	30,349,044	86.88%	477,743	25.74	40.52%
YY1	34,931,138	30,638,355	87.71%	396,032	33.86	43.77%
YY2	34,931,138	31,073,597	88.96%	351,337	35.68	40.35%
YY3	38,529,403	27,913,912	72.45%	471,656	23.49	39.69%
YY4	38,529,403	35,650,291	92.53%	475,029	30.21	40.26%
YY5	38,529,403	35,505,964	92.15%	397,918	39.45	44.21%
YY6	38,529,403	35,633,693	92.48%	358,356	41.28	41.51%
YY7	34,247,393	24,950,345	72.85%	452,411	21.44	38.87%
YY8	34,247,393	31,346,675	91.53%	464,617	28.01	41.52%
YY9	34,247,393	31,407,732	91.71%	376,159	35.89	42.98%
YY10	34,247,393	31,344,967	91.53%	370,976	35.98	42.58%
NJ1	37,946,278	27,287,155	71.91%	469,349	22.39	38.51%
NJ2	37,946,278	34,619,443	91.23%	485,343	28.88	40.48%
NJ3	37,946,278	34,391,689	90.63%	409,869	36.45	43.44%
NJ4	37,946,278	34,647,627	91.31%	384,045	37.55	41.62%
NJ5	29,605,050	22,036,046	74.43%	457,152	18.97	39.36%
NJ6	29,605,050	27,411,265	92.59%	435,252	25.02	39.73%
NJ7	29,605,050	27,191,439	91.85%	375,109	31.68	43.70%

Continue

NJ8	29,605,050	27,199,466	91.87%	348,583	31.83	40.80%
NJ9	28,218,564	20,344,581	72.10%	439,134	18.16	39.19%
NJ10	28,218,564	25,705,754	91.10%	446,689	23.28	40.45%
QZ1	28,218,564	25,731,188	91.19%	366,262	29.84	42.47%
QZ2	28,218,564	26,222,316	92.93%	347,100	31.82	42.13%
QZ3	34,280,064	24,644,212	71.89%	468,670	20.80	39.55%
QZ4	34,280,064	30,964,743	90.33%	476,107	26.88	41.34%
QZ5	34,280,064	31,326,631	91.38%	387,776	35.68	44.17%
QZ6	34,280,064	30,395,369	88.67%	366,284	34.56	41.65%
QZ7	23,684,340	17,027,353	71.89%	421,180	16.11	39.85%
QZ8	23,684,340	21,693,596	91.59%	429,558	20.57	40.72%
QZ12	23,684,340	21,539,011	90.94%	355,515	26.17	43.20%
QZ17	23,684,340	21,617,962	91.28%	324,008	28.25	42.34%
WX3	40,680,935	27,780,743	68.29%	482,999	22.82	39.67%
WX10	40,680,935	34,724,943	85.36%	492,465	28.38	40.25%
WX12	40,680,935	36,439,682	89.57%	441,770	35.12	42.58%
WX24	40,680,935	31,567,061	77.60%	426,260	30.58	41.29%
SD23	25,995,105	18,573,739	71.45%	426,139	16.61	38.11%
SD33	25,995,105	21,390,866	82.29%	446,959	18.86	39.40%
SD37	25,995,105	21,791,494	83.83%	378,680	24.48	42.53%
SD41	25,995,105	23,570,555	90.67%	332,243	28.33	39.94%
SD49	26,900,209	18,282,509	67.96%	442,104	15.27	36.93%
SD50	26,900,209	22,800,733	84.76%	453,697	20.27	40.33%
HK3	26,900,209	23,813,338	88.52%	370,601	27.88	43.39%
HK5	26,900,209	23,894,753	88.83%	333,350	29.83	41.61%
SH12	31,293,809	22,610,746	72.25%	437,860	20.53	39.76%
SH15	31,293,809	25,588,150	81.77%	457,039	22.37	39.95%
SH32	31,293,809	26,917,251	86.01%	430,418	26.24	41.95%
SH35	31,293,809	27,863,730	89.04%	392,583	28.69	40.42%
SH36	28,282,480	20,079,095	70.99%	426,234	19.67	41.76%
SH38	28,282,480	24,734,759	87.46%	462,909	21.87	40.93%
SH41	28,282,480	24,004,253	84.87%	414,658	24.67	42.61%
SH48	28,282,480	24,241,429	85.71%	393,426	25.65	41.63%
WX25	30,522,639	26,469,887	86.72%	477,065	22.54	40.62%
WX27	30,522,639	26,870,036	88.03%	468,930	23.36	40.77%
WX28	30,522,639	26,928,029	88.22%	426,411	27.20	43.07%
WX41	30,522,639	24,793,199	81.23%	391,960	25.23	39.89%
gy23	32,451,303	27,236,337	83.93%	471,123	22.80	39.43%
gy24	32,451,303	27,080,708	83.45%	476,891	22.59	39.79%
gy28	32,451,303	27,424,283	84.51%	433,505	27.55	43.54%
gy29	32,451,303	27,237,515	83.93%	412,002	27.09	40.97%
WX42	50,617,112	43,103,905	85.16%	525,374	32.24	39.30%
WX47	50,617,112	42,560,648	84.08%	511,136	34.21	41.08%

Continue

gy6	50,617,112	41,514,248	82.02%	473,231	38.02	43.34%
gy14	50,617,112	40,884,647	80.77%	453,452	37.18	41.24%
gy31	40,433,096	31,753,877	78.53%	494,324	24.76	38.55%
gy37	40,433,096	34,501,409	85.33%	492,627	27.91	39.85%
gy45	40,433,096	35,987,384	89.00%	442,822	35.41	43.57%
gy46	40,433,096	33,541,662	82.96%	424,545	32.48	41.11%
LZ1	30,621,489	25,075,348	81.89%	468,653	20.48	38.27%
LZ4	30,621,489	27,059,958	88.37%	462,945	23.36	39.97%
LZ5	30,621,489	26,626,082	86.95%	434,713	26.32	42.97%
LZ6	30,621,489	23,188,907	75.73%	391,166	24.71	41.68%
LZ10	43,394,955	31,628,332	72.88%	487,973	25.44	39.25%
LZ11	43,394,955	37,828,599	87.17%	495,799	31.55	41.35%
LZ20	43,394,955	35,521,682	81.86%	455,552	32.72	41.97%
LZ24	43,394,955	37,399,966	86.19%	437,925	35.46	41.52%
LZ28	30,714,387	26,287,995	85.59%	491,893	21.72	40.64%
LZ33	30,714,387	26,413,728	86.00%	472,120	22.55	40.30%
XY3	30,714,387	24,729,466	80.51%	431,917	23.98	41.88%
XY4	30,714,387	25,769,443	83.90%	412,174	25.57	40.90%
XY14	31,822,767	27,115,110	85.21%	486,776	22.18	39.82%
XY15	31,822,767	27,381,403	86.04%	476,621	22.65	39.42%
XY23	31,822,767	28,110,482	88.33%	417,546	28.15	41.81%
XY32	31,822,767	25,255,602	79.36%	415,418	25.54	42.00%
XY33	25,871,265	21,962,454	84.89%	466,904	18.48	39.28%
XY36	25,871,265	22,569,168	87.24%	448,343	19.55	38.84%
FH013	25,871,265	18,893,935	73.03%	379,986	20.31	40.84%
XY44	25,871,265	22,280,544	86.12%	385,293	23.69	40.96%
SD3	40,803,146	35,802,193	87.74%	495,263	27.45	37.98%
SD4	40,803,146	33,127,059	81.19%	491,381	26.86	39.84%
SD9	40,803,146	35,915,492	88.02%	398,321	38.25	42.42%
SD21	40,803,146	35,731,346	87.57%	372,655	38.30	39.94%
HK9	44,271,578	38,139,538	86.15%	514,107	29.65	39.97%
HK11	44,271,578	38,345,890	86.62%	492,470	31.71	40.72%
HK15	44,271,578	36,577,341	82.62%	423,302	38.31	44.34%
HK30	44,271,578	34,539,572	78.02%	394,467	38.26	43.70%
HK36	31,639,345	27,391,819	86.58%	486,108	23.03	40.87%
HK44	31,639,345	27,318,029	86.34%	466,327	24.20	41.31%
HK46	31,639,345	25,599,252	80.91%	402,202	27.40	43.05%
HK48	31,639,345	27,632,693	87.34%	361,991	32.27	42.27%
HZ1	33,504,364	28,330,245	84.56%	501,680	22.35	39.58%
HZ2	33,504,364	28,692,933	85.64%	474,135	24.91	41.17%
HZ3	33,504,364	28,764,421	85.85%	402,098	31.60	44.17%
HZ5	33,504,364	27,351,108	81.63%	379,561	31.58	43.82%
HZ8	26,077,592	22,083,138	84.68%	464,438	18.49	38.89%

Continue

HZ19	26,077,592	21,874,842	83.88%	434,045	21.28	42.22%
HZ28	26,077,592	19,355,573	74.22%	367,298	23.54	44.66%
HZ29	26,077,592	20,859,944	79.99%	346,697	25.25	41.97%
HZ50	25,877,764	19,773,010	76.41%	452,958	16.17	37.05%
HZ52	25,877,764	21,646,343	83.65%	451,505	19.10	39.83%
SH3	25,877,764	22,048,303	85.20%	367,293	26.22	43.68%
SH11	25,877,764	22,067,694	85.28%	334,863	27.36	41.51%

Table S2 Geographic information of 22 sample sites

Location	Abbreviation	Latitude	Longitude	Date
Yueyang Hunan	YY	N29°09′29.62″	E113°0′4.98″	2016.04.11
Nanjing Jiangsu	NJ	N32°19′27.95″	E118°48′58.93″	2016.04.16
Quzhou Zhejiang	QZ	N29°08′19.27″	E118°24′32.41″	2016.04.21
Huangshan Anhui	HS	N29°48′0.08″	E118°12′22.77″	2016.04.22
Weishan lake Shongdong	SD	N35°01′2.89″	E116°56′12.74″	2016.07.22
Hukou Jiangxi	HK	N29°46′16.55″	E116°20′17.32″	2016.07.27
Hangzhou Zhejiang	HZ	N30°16′16.53″	E120°03′45.32″	2016.08.02
Wuxi Jiangsu	WX	N31°26′9.66″	E120°15′35.10″	2016.08.05
Chongming Shanghai	SH	N31°43′43.35″	E121°13′36.97″	2016.08.05
Gaoyou Jiangsu	GY	N32°49′43.87″	E119°30′32.89″	2016.08.06
Laozi Anhui	LZ	N33°11′7.68″	E118°35′25.34″	2016.08.07
Xuyu Jiangsu	XY	N33°02′32.98″	E118°28′57.78″	2016.08.08
Pizhou Jiangsu	PZ	N34°19′47.45″	E117°54′1.04″	2016.08.10
Honghu Hubei	HH	N29°40′11.32″	E113°19′0.77″	2016.08.13
Feihe Anhui	FH	N33°09′55.34″	E117°06′53.00″	2016.08.14
Wusheng Sichuan	WS	N30°20′41.54″	E106°16′26.37″	2016.08.18
Yibin Sichuan	YB	N28°47′41.22″	E104°41′9.94″	2016.08.19

Zhongxian Chongqing	CQ	N30°18′9.86″	E108°01′59.41″	2016.08.23
Xinyang Henan	HN	N32°04′38.46″	E114°02′39.74″	2016.08.23
Danjiangkou Hubei	DJK	N32°32′33.47″	E111°30′26.31″	2016.08.25
Yichang Hubei	YC	N31°45′4.75″	E112°30′37.74″	2016.08.28
Jizhou Hubei	JZ	N30°13′37.41″	E112°07′3.17″	2016.08.28
Liangzi lake Hubei	LZH	N30°10′1.59″	E114°37′46.95″	2016.08.30
